# RETRACTION: Visualization of the Relationship Between Macrophage and Wound Healing From the Perspective of Bibliometric Analysis

**DOI:** 10.1111/iwj.70670

**Published:** 2025-04-23

## Abstract

**RETRACTION:** GuoQ. , LiW. , XieR. , WangY. , XieY. , ChengK. , and SunZ. , “Visualization of the Relationship Between Macrophage and Wound Healing From the Perspective of Bibliometric Analysis,” International Wound Journal 21, no. 4 (2024): e14597, 10.1111/iwj.14597.38124467 PMC10961877

The above article, published online on 20 December 2023, in Wiley Online Library (http://onlinelibrary.wiley.com/), has been retracted by agreement between the journal Editor in Chief, Professor Keith Harding; and John Wiley & Sons Ltd. Following an investigation by the publisher, all parties have concluded that this article was accepted on the basis of a compromised peer review process. In addition, further investigation by the publisher found that this article is missing the underlying dataset, which contradicts the Data Availability Statement. The investigation found that the Methods and Results included irrelevant citations or otherwise lacked proper citations, which leaves large sections of the statements made unsubstantiated by the academic literature. Lastly, the investigation found that the statistical analysis was incomplete. The editors have therefore decided to retract the article. The authors did not respond to our notice regarding the retraction.

**RETRACTION:** Q. Guo , W. Li , R. Xie , Y. Wang , Y. Xie , K. Cheng , and Z. Sun , “Visualization of the Relationship Between Macrophage and Wound Healing From the Perspective of Bibliometric Analysis,” International Wound Journal 21, no. 4 (2024): e14597, 10.1111/iwj.14597.38124467 PMC10961877

The above article, published online on 20 December 2023, in Wiley Online Library (http://onlinelibrary.wiley.com/), has been retracted by agreement between the journal Editor in Chief, Professor Keith Harding; and John Wiley & Sons Ltd. Following an investigation by the publisher, all parties have concluded that this article was accepted on the basis of a compromised peer review process. In addition, further investigation by the publisher found that this article is missing the underlying dataset, which contradicts the Data Availability Statement. The investigation found that the Methods and Results included irrelevant citations or otherwise lacked proper citations, which leaves large sections of the statements made unsubstantiated by the academic literature. Lastly, the investigation found that the statistical analysis was incomplete. The editors have therefore decided to retract the article. The authors did not respond to our notice regarding the retraction.

